# 

**Published:** 1881-06

**Authors:** 


CONTENTS:

| | | Page. |
|---|---|---|
| ***ORIGINAL.*** | | |
| The Inter-National Hahnemannian Association, | Editorial, | 223 |
| Fatal Errors, | Ad. Lippe, M.D., Phila. | 230 |
| The Question Answered, | C. Carleton Smith, M.D., Phila. | 232 |
| Fluxion Dilutions, | Wm. Jefferson Guernsey, M.D., Phila. | 236 |
| Diphtheria and Bacteria, | P. P. Wells, M.D., Brooklyn, N.Y. | 239 |
| Instruction not Endorsement, | T. F. Pomeroy, M. D., Jersey City, | 252 |
| ***CLINICAL CASES:*** | | |
| Cases of Chronic Disease—cured, | Thos. Skinner, M.D., Liverpool. | 254 |
| Periscope, | | 259 |
| ***BOOK NOTICES AND REVIEWS.*** | | |
| Diagnosis of Disease, &c., | Richard Hagen, M.D., | 270 |
| Biliary Calculi, &c., | Von Tagen, M.D., | 270 |
| Spectacles and How to Choose Them, | C. A. Vilas, M. D., | 271 |
| Surgical Principles and Minor Surgery, | J. G Gilcheist, M. D., | 271 |
| Prevention of Congenital Malformations, &c., | J. C. Burnett, M D., | 272 |
| How to See with the Microscope, | J. Edwards Smith, M.D., | 272 |

